# 

**Published:** 1903-11-21

**Authors:** 


**CADBURY'S COCOA** "A PERFECT FOOD."

**CADBURY'S COCOA** NO DRUGS OR ALKALIES USED.

### THE HOSPITAL

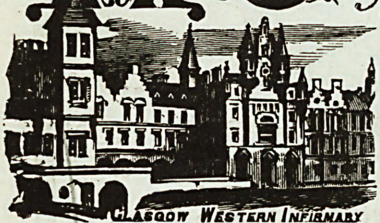

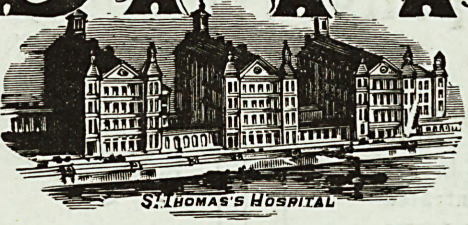

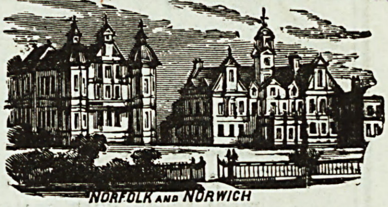

No. 895. Vol. XXXV. [Registered as a Newspaper.] SATURDAY, NOV. 21ST, 1903. [Subscription Terms, see p. 128.] PRICE THREEPENCE.

